# Supplementary material for: A general method for controlling and resolving rotational orientation of molecules in molecule-surface collisions
Source: Nat Commun. 2017 May 8;8:15357. doi: 10.1038/ncomms15357 (PMC5424257; doi:10.1038/ncomms15357)
Supplement: Supplementary Information — Supplementary Figures, Supplementary Tables, Supplementary Notes and Supplementary References [file ncomms15357-s1.pdf]

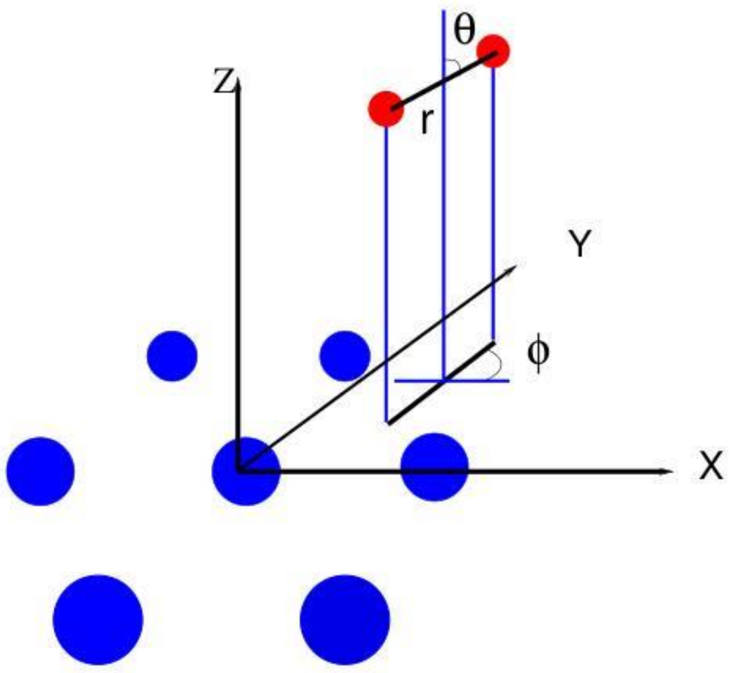

Supplementary Figure 1. The coordinates used for H_2 + Cu(111) calculations. *X*, *Y*, *Z* mark the center of mass coordinates and *r*, *θ* and *Φ* mark the H-H inter-nuclear distance and the molecule's orientation relative to the surface.

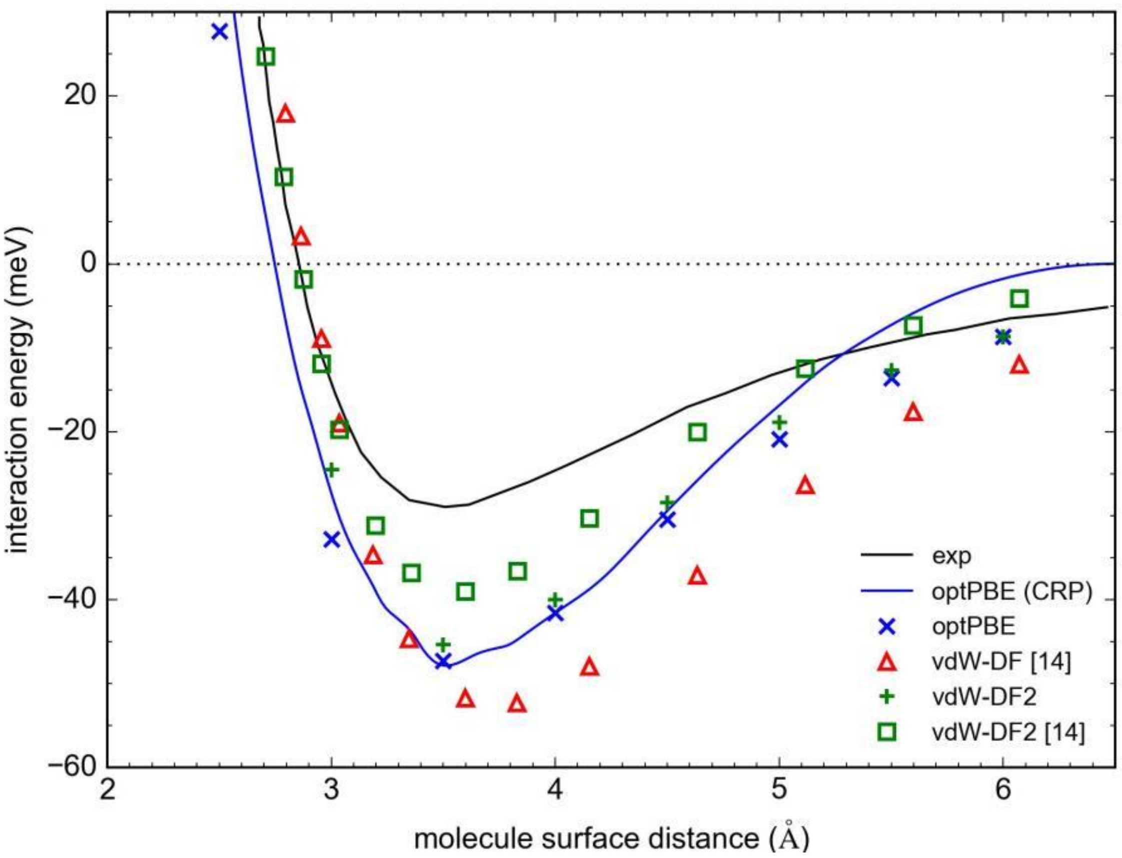

Supplementary Figure 2. H_2-Cu(111) interaction is shown as a function of *Z* (in Å). The interaction is plotted for H_2 approaching a top site along the line normal to the surface, with H_2 held parallel to the surface and oriented towards the two neighboring bridge sites. Results obtained with the optPBE-vdW (blue crosses) and vdW-DF2 functionals (green pluses) are from this work, as well as those calculated based on the continuous optPBE-vdW (CRP) PES representation (blue line). Results obtained from experiments (black line), and previous calculations with the vdW-DF (red triangles) and vdW-DF2 (green squares) functional are also shown; these results were obtained from Ref.[1] (see also the text).

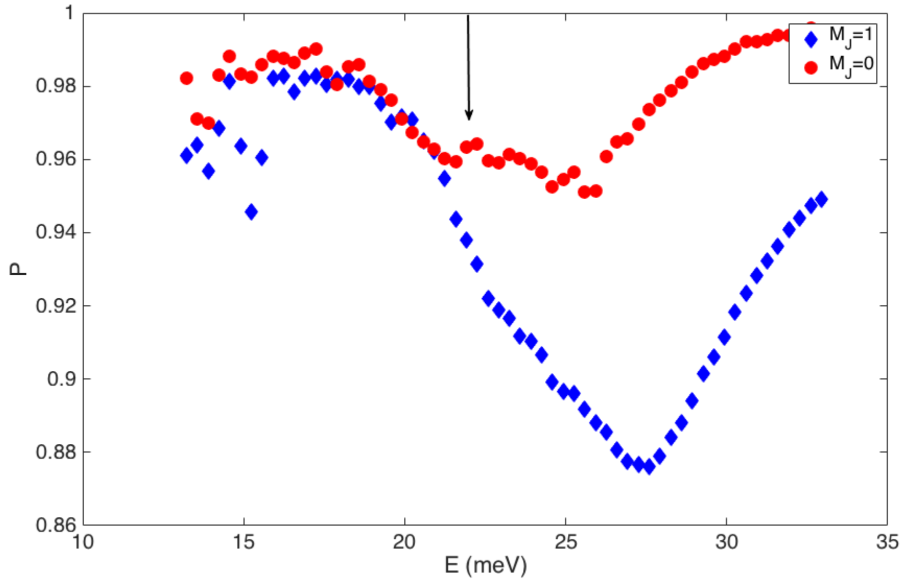

Supplementary Figure 3. **Calculated probabilities of specular scattering for different m_J states.** The probabilities are shown for m_J=0 and m_J=1 (or, equivalently, m_J=-1) molecules as a function of total incidence energy. The arrow marks the experimental conditions.

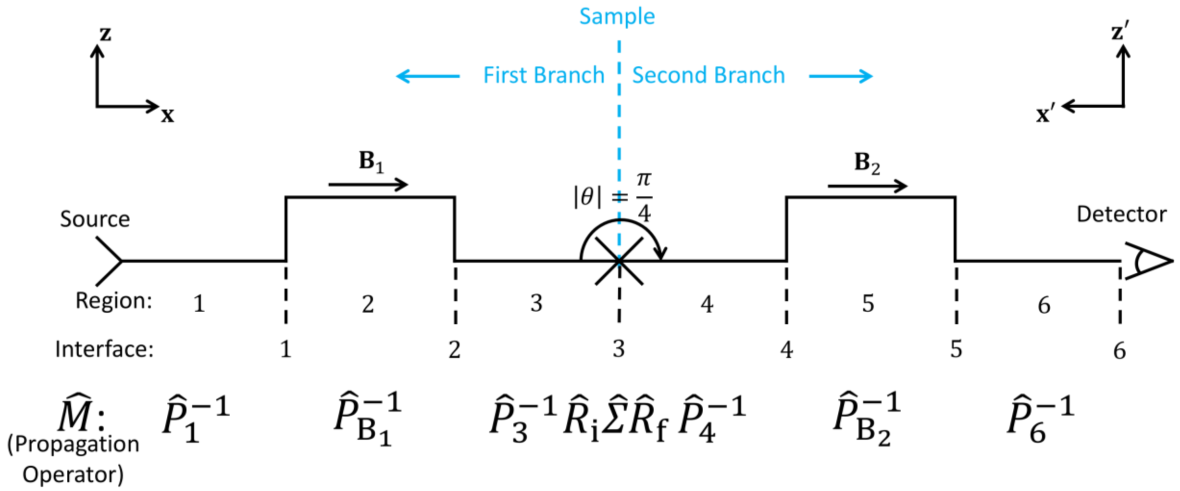

Supplementary Figure 4. Schematic diagram depicting different propagation regions of the two-arm apparatus. $\hat{\boldsymbol{M}}$ is an overall propagation operator that includes the operators specified below each propagation region (see Eq. S8). These operators are defined such that they operate on the initial state to retrieve the final state. $\mathbf{B}_i$ refers to the different magnetic field vectors, **z**' and **x**' refer to the new coordinate system defined to align with the second branch of the apparatus. The sample is located at interface 3. Regions 1, 3, 4 and 6 are field-free. The propagation direction is **x** before scattering and –**x**' after scattering. The angle between **x** and –**x**' (i.e. the angle between the two arms of the apparatus) is θ = 45°. The magnetic field is directed along **x** in region 2 and along –**x**' in region 5. The first and second hexapole lenses are incorporated into the source and detector, respectively, in this diagram.

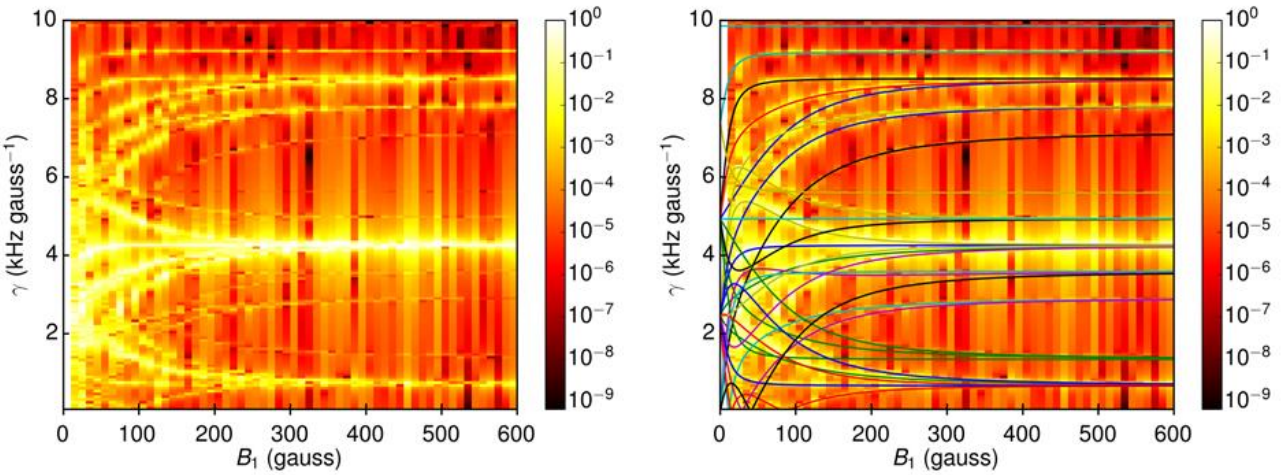

Supplementary Figure 5. 2D Spectrum plot obtained from the theoretical model. The calculation was performed for a statistical mixture defined as $\boldsymbol{\rho}(\boldsymbol{k_0}) = \sum_{m_I m_J} \boldsymbol{\eta}_{m_I m_J} |\boldsymbol{k_0 m_I m_J}\rangle\langle\boldsymbol{k_0 m_I m_J}|$ (left panel). Same spectrum overlaid with lines corresponding to the generalized gyromagnetic ratios of $\widehat{\boldsymbol{H}}_{\mathbf{R}}$ (right panel).

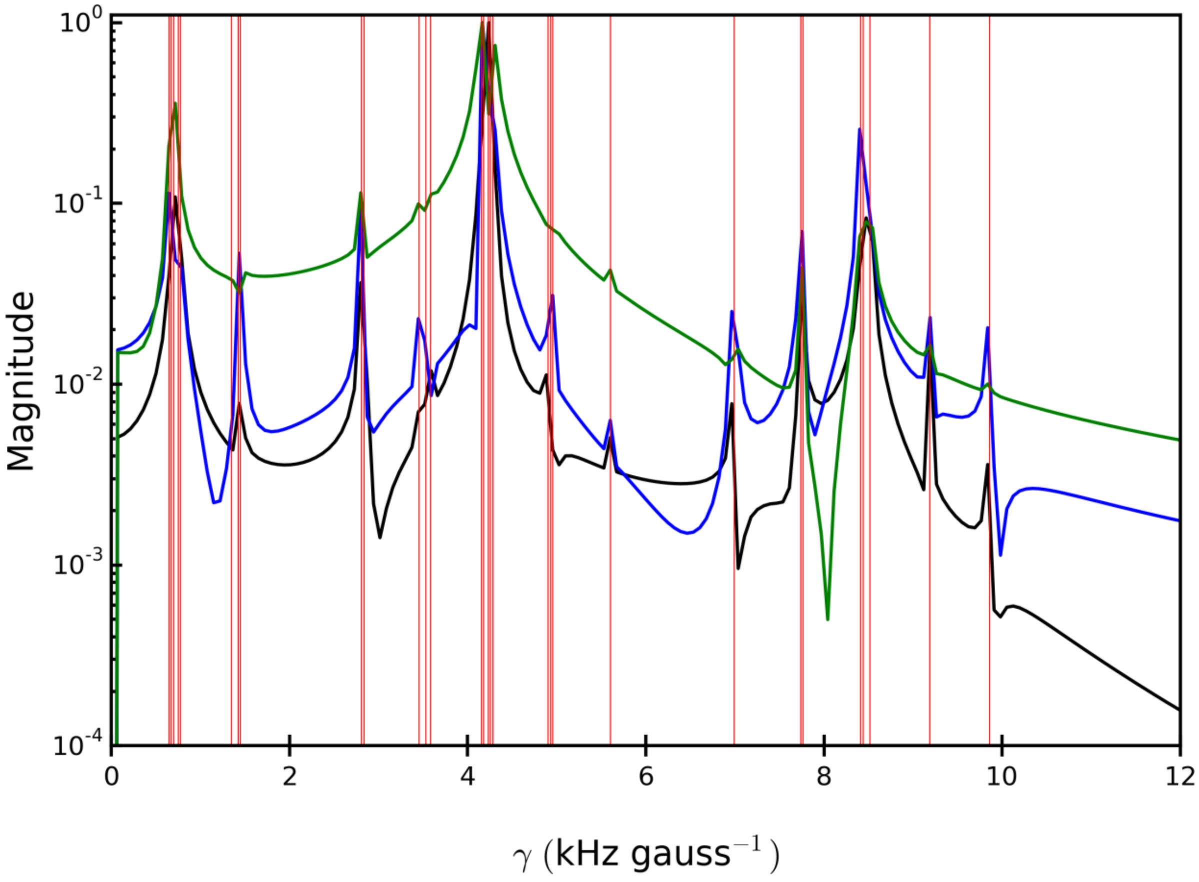

Supplementary Figure 6. Demonstrating the effect of a phase change during the scattering event. Spectra obtained for calculations with no phase changes due to scattering (black) and random phases for each scattered state (blue and green). Phases were chosen from a uniform distribution ranging from 0 to 2π. Vertical red lines mark the positions of the Ramsey transitions. B_1 is 400 gauss, B_2 is varied from -410 to -390 gauss, the initial state is $(\boldsymbol{m_I}, \boldsymbol{m_J}) = (\mathbf{1}, \mathbf{1})$, and the central velocity is 1436.14 m s^{-1}.

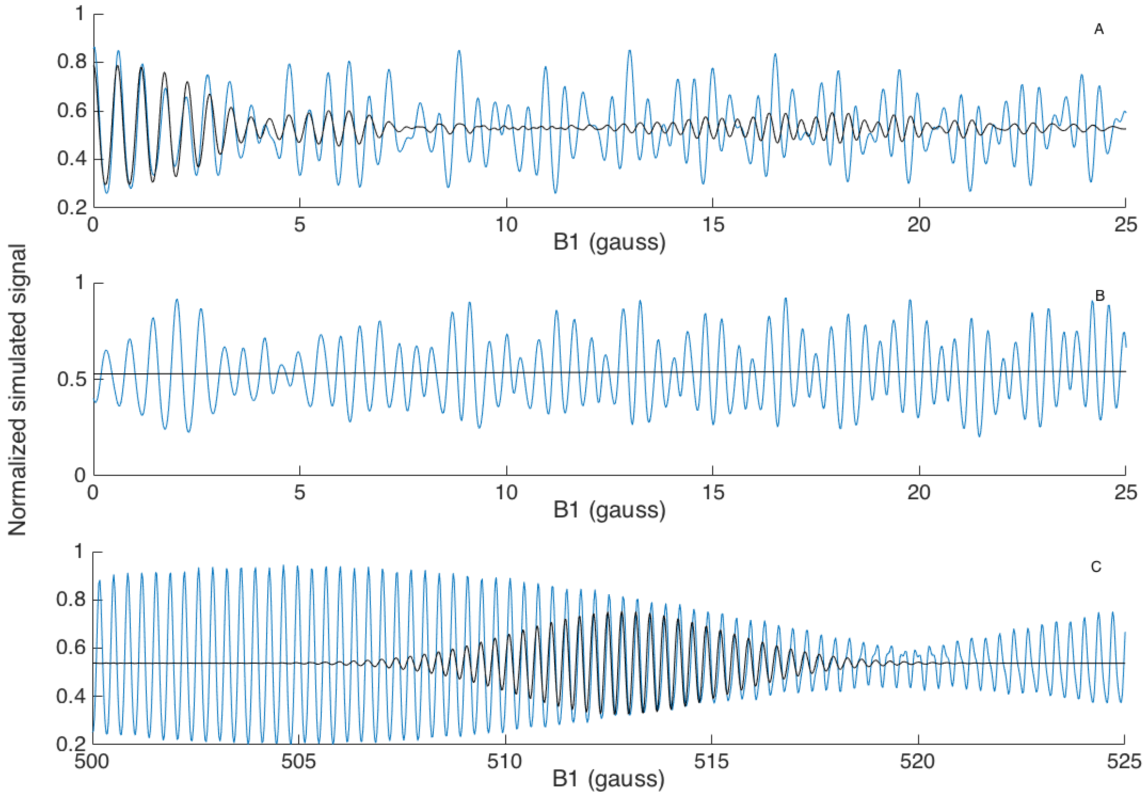

Supplementary Figure 7. Calculation of the detected signal for balanced and unbalanced field conditions. The calculations were performed for a beam which does not undergo any change in probability or quantum state during the scattering event. Blue lines are for a single velocity and black lines are after averaging over the velocity distribution of the beam. (A) Scanning B_1 close to 0 when B_2=0 leads to oscillations in the signal which are related to the quantum state entering the B_2 field, i.e. (full interferometer mode). (B) Performing the same B_1 scan for a fixed B_2=500 gauss value produces no oscillations in the beam averaged signal due to the depolarization effect mentioned above and the signal is constant (flux detection mode). (C) When B_1 is scanned close to the value of -B_2, the velocity spread is compensated and an oscillating echo is formed (full interferometer mode).

Supplementary Table 1. The parameters used for the quantum dynamics calculations on H_2 on Cu(111).

Parameter	Value
# points in Z	180
# points in Z, specular grid	384
Start of Z grids	-1.0 a_0
Step size of grids in Z	0.2 a_0
# points in r	64
Start of grid in r	0.4 a_0
Step size of grid in r	0.15 a_0
# points in X (Y)	16
Lattice constant	2.5805 a_0
Maximum J in angular basis set	7
Maximum m_J in angular basis set	7
Time step for propagation	0.0605 fs
Total propagation time	6.228 ps
Location of analysis line in Z	9.2 a_0
Location of analysis line on specular Z grid	34.6 a_0
Location of the initial wave packet in Z	22.0 a_0
Normal incidence range in Z	10 meV to 30 meV
Initial parallel incidence energy along the	3.689 meV
Range of optical potential on Z grid	9.2 a_0 to 34.8 a_0
Strength of optical potential on Z grid	0.0044 E_h a_0^{-2}
Range of optical potential on specular Z grid	34.8 a_0 to 75.6 a_0
Strength of optical potential on specular Z	0.0096 E_h a_0^{-2}
Range of optical potential on r grid	4.2 a_0 to 8.2 a_0
Strength of optical potential on r grid	0.0092 E_h a_0^{-2}

Supplementary Table 2. Relative transmitted state probabilities of the quantum state selectors. $\boldsymbol{\eta_{m_I m_J}}$ $\boldsymbol{c_{m_I m_J}}$ mark the transmission probabilities for the first (polarizer) and second (analyzer) hexapole magnets.

m_I	1	1	1	0	0	0	-1	-1	-1
m_J	1	0	-1	1	0	-1	1	0	-1
$\eta_{m_I m_J}$	1.0000	0.9755	0.7901	0.1465	0.1111	0.0738	0.0343	0.0299	0.0258
$c_{m_I m_J}$	1.00	0.96	0.93	0.53	0.42	0.37	0.21	0.19	0.16

Supplementary Note 1. Density functional theory calculations for H_2 on Cu(111) and Cu(511).

Density functional theory (DFT) calculations have been carried out with the plane-wave DFT code VASP. The slab model for the Cu(111) (Cu(511)) surface consisted of a 2x2 (a 1x2) multiple of the surface unit cell, using a vacuum distance of 15 A and an 8x8 (a 6x8) Gamma-centered *k*-point grid for the Brillouin zone sampling. Further computational settings (standard PAW potentials, 400 eV cut-off energy, Gaussian smearing with a width of 0.1 eV) have been chosen identical to those in Ref. [2]

For the potential energy curves (PEC) shown in Fig.3 of the main article, the H_2 molecule has been placed over a top site. At each point along these curves, the H-H bond distance was allowed to relax. The molecular axis is tilted relative to the (macroscopic) surface normal, i.e. the [111] and [511] direction for Cu(111) and Cu(511), respectively, so that both hydrogen atoms are pointing towards the two neighboring bridge sites. In the molecular coordinate system used for Cu(111) shown in Supplementary Fig. 1, this corresponds to X=0, Y=0, and Φ=0 (varying Z and r for different values of θ). For Cu(511), the molecular axis is parallel to the step edge. The few other orientations that have been tried for this surface did not yield notably different results.

A particular focus has been put on the optPBE-vdW density functional[3]. This functional offers an accurate description of reactive scattering of H_2 from Cu(111)[2], and ensures a chemically accurate description of the van der Waals interaction in van der Waals molecules, as established for the S22 database[3]. Supplementary Fig. 2 shows a comparison of potential curves obtained

with different functionals for H_2 interacting with Cu(111). As can be seen, calculations with the optPBE density functional describe the van der Waals interaction determined experimentally semi-quantitatively, with the potential minimum approximately in the right place, albeit that the well depth is overestimated somewhat (by about 20 meV). As can also be seen in Supplementary Fig. 2, the optPBE-vdW density functional yields a better description of the van der Waals interaction than the vdW-DF functional of Dion et al.[4] Within our computational setup we find that optPBE-vdW performs equally well as the vdW-DF2 functional.[5] This functional has been used for the calculation of interaction energies of H_2 with Cu(111) before[6], but it has not yet been tested on reactive scattering.

Supplementary Note 2. Quantum dynamics calculations of probabilities for H_2+Cu(111) scattering

We use the Born-Oppenheimer static surface (BOSS) model for the calculation of the S-matrix and the probabilities for specular scattering of (v=0, J=1, m_J) H_2 from Cu(111). The molecule-surface interaction was calculated within the Born-Oppenheimer approximation[7], using density functional theory (DFT)[8,9]. Furthermore, the atoms were kept fixed in their ideal lattice positions. Within the BOSS model, energy transfer to electron-hole pair excitation and the surface phonons is not allowed. As discussed in for instance Ref.[2], these approximations allow accurate results to be obtained for reactive and non-reactive scattering of H_2 from metal surfaces, as also shown specifically for one of the systems studied in the present work, i.e., H_2 + Cu(111)[10].

Within the BOSS model, the scattering dynamics in the remaining six molecular degrees of freedom of hydrogen can be treated without any further approximations using quantum dynamics[6,11–13]. The coordinates used for the six molecular degrees of freedom are shown in Supplementary Fig. 1: X, Y, and Z are the center-of-mass coordinates describing translational motion parallel and normal (Z) to the surface, and r, θ and Φ are the coordinates describing the H-H inter-nuclear distance, the polar angle of orientation with respect to the Z-axis, and the azimuthal angle of orientation. We perform the scattering calculations in this coordinate frame to obtain the S-matrix elements giving the probability amplitudes for transitions between the rotational states (J, m_J), with m_J specifying the eigenstates of the projection of the rotational angular momentum on the *normal to the surface*. For the scattering calculations, we neglect the hyperfine interaction. Since the molecules are prepared as coherent superpositions of the eigenstates (F, m_F) of the full molecular Hamiltonian (including the hyperfine interaction) with the quantization axis set by the direction of the magnetic field before they arrive at the surface, we need to rotate the coordinate system and project the resulting states onto the states with projections m_J in the coordinate frame of the scattering calculations. These coordinate rotations are described in the next section.

We use a potential energy surface (PES) computed with the optPBE-vdW density functional[3], for the reasons already discussed in Section 1. As described in detail in Ref. [2], DFT calculations performed on a grid of points were interpolated using the accurate corrugation reducing procedure (CRP)[14,15], which also yields an accurate description of the molecule-surface interaction for points not included in the database for interpolation. Full details are presented in Ref.[2].

To obtain dynamical results, we use the time-dependent wave packet (TDWP) method[16] in an implementation describing a diatomic molecule scattering from a surface with hexagonal symmetry[17]. A Gaussian-shaped wave packet in Z is prepared to describe the hydrogen approaching the surface for a normal incidence energy range of 10 to 30 meV. The initial parallel momentum along the <11-2> direction of incidence (i.e., the selected XZ plane) was chosen to be 3.21 meV, such that at a normal incidence energy of 18.74 meV (and a total incidence energy of 21.95 meV) the experimental polar angle of incidence (22.5°) was obtained. The hydrogen molecule is prepared in its initial rovibrational (v=0,J=1,m_J=-1,0 or 1) state, and a wave packet propagation is performed for all three initial states. The scattering amplitude formalism [18,19] is used to retrieve a column of the S-matrix for the scattering from the initial state (v,J,m_J) into the final states (v',J',m_J',n,m), with n and m being the diffraction quantum numbers. Specular scattering corresponds to scattering into the (v'=v,J'=J,m_J',n=0, m=0) final states, and for the flux sensitive detector used in the experiments, for a pure initial m_J state the probability for specular scattering can be obtained by summing over m_J' according to

$$P(v=0, J=1, m_J \rightarrow v'=0, J'=1, n=0, m=0) = \sum_{m_J'=-1}^{1} P(v=0, J=1, m_J \rightarrow v'=0, J'=1, m_J', n=0, m=0) \quad (S1)$$

Supplementary Fig. 3 shows the calculated specular scattering probabilities of m_J=0 and m_J=1 initial states.

Note that for rotationally elastic (J'=J), specular scattering (n=m=0) of J=1 H_2 of Cu(111), the threefold rotational symmetry of Cu(111) dictates that m_J is conserved (m_J'= m_J), and the S-matrix elements (for a given energy value) obey:

$$S_{v=0,J=1,m_J=1\rightarrow v'=v,J'=J,m_J'=m_J,n=0,m=0} = S_{v=0,J=1,m_J=-1\rightarrow v'=v,J'=J,m_J'=m_J,n=0,m=0} \quad \text{(S2)}$$

The projection of the nuclear magnetic angular momentum on the Z-axis, m_I, is assumed to be conserved during the short time H_2 interacts with the surface. As a result, the block of the S-matrix corresponding to J=1 is diagonal in both m_I and m_J.

The wave function is described with a discrete variable representation (DVR, [20], i.e., Fourier grids) in r, X, Y and Z. For the angular degrees of freedom a finite basis representation (FBR) in J and m_J of spherical harmonics was chosen. To transform the angular FBR to the corresponding DVR in order to apply the potential energy operator, Gauss-associated Legendre transforms were used[21,22]. The propagation of the wave packet was done using the Split-Operator formalism[23]. Suitable grids in Z and r were chosen to also accommodate quadratic optical potentials to absorb the returning wavepacket[24]. To reduce the computational effort a special grid in Z for the specular channel was used[17]. This allowed us to use an extra-long initial Z grid to represent the energetically narrow initial wave packets for the low incidence energies employed. The actual parameters used in these calculations are shown in Supplementary Table 1.

Supplementary Note 3. Theoretical simulation of the two-arm molecular interferometer

Time independent approach

In order to simulate the evolution of molecular wavepackets before and after the scattering event, we solve the Schrodinger equation. We account for changes of phases and momenta in each component of the molecular wave packets; these changes are due to gradients of magnetic fields and regions of static field throughout the apparatus. We assume that the ortho (*J*=1) H_2 molecules are thermalized before the supersonic expansion and well-described by the density matrix

$$\rho = \sum_{Fm_F} p_{kFm_F} |kFm_F\rangle\langle kFm_F| \qquad (S3)$$

where *k* is the linear momentum of the H_2 molecules in the *x*-direction and *x* is the incident beam propagation direction before molecules arrive at the surface (see Supplementary Fig. 4 for the definition of the coordinate systems and operators propagating molecules through various stages of the apparatus before and after scattering). In Eq. S3, *F* is the total angular momentum (a vector sum of the nuclear *I* and rotational *J* angular momenta of oH_2), and m_F is the projection of the angular momentum *F* onto the *z*-axis directed perpendicular to the propagation direction, as shown in Supplementary Fig.4. The coefficients p_{kFm_F} follow the Boltzmann distribution with the Hamiltonian

$$\hat{H} = \frac{\hbar^2 k^2}{2m_{\mathrm{H}_2}} + \hat{H}_{\mathrm{R}}(\mathbf{B} = 0) \qquad (S4),$$

where $\hat{H}_{\mathrm{R}}(\mathbf{B})$ is the Ramsey Hamiltonian defined in [25], $\hbar$ is the reduced Planck constant, m_{H_2} is the H_2 mass and **B** is the magnetic field. Note that the magnetic field in the gas before its supersonic expansion is that of the Earth and near zero (<0.65 gauss), such that the Fm_F states are a very good approximation to the eigenstates.

The probabilities can be written as $p_{kFm_F} = \beta_k \alpha_{Fm_F}$, since $[k^2, \hat{H}_{\mathrm{R}}] = 0$, where β_k and α_{Fm_F} follow the Boltzmann distributions for the k^2 and $\hat{H}_{\mathrm{R}}$ terms of the above Hamiltonian, respectively. The first hexapole magnet (labeled as magnetic lens 1 in Fig.1a) preferentially selects particular states in the $|m_I m_J\rangle$ basis, changing the probabilities: $p_{kFm_F} \to \beta_k \eta_{m_I m_J}$. The values of $\eta_{m_I m_J}$ are determined via semi-classical simulations of the molecular propagation through the hexapole magnet (see values in Supplementary Table 2 calculated using the ray tracing procedure described in Ref. [26]) and m_I and m_J are the projections of I and J onto the z-axis of Supplementary Fig. 4, respectively. As Supplementary Table 2 shows and discussed in the main text, the hexapole magnet mainly transmits molecules with (m_I, m_J) = (1,1), (1,0), and (1,-1).

We assume that the distribution in k, with a FWHM of 4%, is well-described by its central momentum $k_0 = \frac{m_{\mathrm{H}_2} v_0}{\hbar}$. Thus, after passing through the hexapole magnet, we can describe the system with

$$\rho(k_0) = \beta_{k_0} \sum_{m_I m_J} \eta_{m_I m_J} |k_0 m_I m_J\rangle\langle k_0 m_I m_J| \qquad \text{(S5).}$$

To model the propagation through the entire apparatus before and after scattering, we use the path coordinate, rotating the coordinate system to account for changes of the magnetic field direction when necessary. This formulation allows us to use a modified transfer matrix formalism[27], with propagators accounting for the changes of the coordinate systems and the magnitudes of the magnetic fields. In particular, the underlying *k*-state is viewed as having nine internal degrees of freedom that are coupled to the external environment via terms in $\widehat{H}_{\mathrm{R}}$. This method is equivalent to solving the time-independent Schrödinger equation in each part of the system and matching the wavefunction coefficients at the boundaries.

The following is a brief general description of the propagation method in a full-interferometer type experiment. Given an arbitrary initial state

$$|\psi(x=0)\rangle = \sum_{m_I m_J} g_{m_I m_J} \left|k_0 m_I m_J\right\rangle \qquad \text{(S6)},$$

we can write

$$|\psi(x_{\mathrm{f}})\rangle = \widehat{M}|\psi(x=0)\rangle \qquad \text{(S7)} \qquad ,$$

where $\widehat{M}$ is an operator that describes propagation through the magnetic fields of the apparatus and x_{f} is the location of the detector. As per the transfer matrix formalism, $\widehat{M}$ is a product of several other operators that describe each segment of the propagation in turn. In this system,

$$\widehat{M} = \widehat{P}_6^{-1}\widehat{P}_{\mathrm{B}_2}^{-1}\widehat{P}_4^{-1}\widehat{R}_{\mathrm{f}}\widehat{\Sigma}\widehat{R}_{\mathrm{i}}\widehat{P}_3^{-1}\widehat{P}_{\mathrm{B}_1}^{-1}\widehat{P}_1^{-1} \qquad \text{(S8)},$$

where $\widehat{P}_i^{-1}$ describes magnetic field-free propagation through region i, $\widehat{P}_{\mathrm{B}_j}^{-1}$describes propagation through magnetic field B_j, and $\widehat{\Sigma}$ describes the scattering off of the sample surface (note that this $\widehat{\Sigma}$ is not identical to the S-matrix, but they are related and can be transformed into one another).

The regions are defined in Supplementary Fig. 4 and correspond to different parts of the apparatus; for example, region 1 is field-free propagation between the magnetic lens 1 and the first region of the magnetic field. The scattering operator is assumed to be defined in the coordinate frame with the z-axis pointing along the normal to the surface (see Section 2); $\hat{R}_{\mathrm{i}}$ and $\hat{R}_{\mathrm{f}}$ are the operators that rotate the basis from the frame of the incident beam to the frame of the scattering calculations, and to the frame of the scattered beam, respectively.

It should be noted that the kinetic energy of the H_2 molecules is large enough that scattering off of the boundaries of the magnetic field is negligible (the reflection coefficient $R \approx 2 \times 10^{-13}$). Thus, the discontinuity matrices used in the transfer matrix formalism to describe this reflection are equal to the identity matrix and are subsequently ignored. Definitions of the operators are as follows:

$$\hat{P}_i^{-1} = \sum_{Fm_F} e^{ik_0 L_i} |k_0 F m_F\rangle\langle k_0 F m_F| \qquad (S9)$$

where L_i is the length of region i. $L_1 = L_6 = 0.1$ m and $L_3 = L_4 = 0.3$ m were used for the model. Furthermore,

$$\hat{\Sigma} = \sum_{Fm_F, F'm_F'} \sigma^{k_0}_{F'm_F'Fm_F} |k_0 F' m_F'\rangle\langle k_0 F m_F| \qquad (S10)$$

where $\sigma^{k_0}_{F'm_F'Fm_F}$ describes the amplitude to scatter from state $|k_0 F m_F\rangle$ to $|k_0 F' m_F'\rangle$. These amplitudes can be obtained from the S-matrix discussed in section 2 using the transformation between the uncoupled and coupled angular momentum bases, the selection rules also discussed in section 2, and the conversion from the scattering matrix formalism to the transfer matrix

formalism. Alternatively, these amplitudes can be treated as free parameters to be determined from fitting to the experimental observations of the interference patterns at the detector.

The propagation through the magnetic field B_j can be described by

$$\hat{P}_{B_j}^{-1}|\psi\rangle = \sum_{F'm_F',R,Fm_F} \langle k_0 F'm_F' | k_{Fm_FR} R\rangle e^{ik_{Fm_FR}L} \langle k_{Fm_FR} R | k_0 F m_F\rangle a_{Fm_F} |k_0 F'm_F'\rangle \quad (S11)$$

where $a_{Fm} = \langle k_0 F m_F|\psi\rangle$, $\hat{H}_{\mathrm{R}}(\mathbf{B})|R\rangle = E_R(\mathbf{B})|R\rangle$, k_{Fm_FR} is the kinetic energy of the $o\mathrm{H}_2$ molecule inside the magnetic field B_1 or B_2 (see Fig.1A) (k_{Fm_FR} is determined by the conservation of energy), and L is the length of each magnetic field (1m). The action of the magnetic field is two-fold. It changes the energies of the states and simultaneously mixes them into the new eigenstates

$$|k_{Fm_FR} R\rangle = \sum_{Fm_F} \langle k_0 F m_F | k_{Fm_FR} R\rangle \, |k_0 F m_F\rangle \qquad (S12).$$

This mixing and the simultaneous change of momentum from $k_0 \to k_{Fm_FR}$, different for each of the 81 pairs of $|Fm_F\rangle$ and $|R\rangle$, makes the simulations nontrivial. $|R\rangle$ is an eigenstate of $\hat{H}_{\mathrm{R}}(\mathbf{B})$ and is also a function of the magnetic field. The 81 pairs arise from all possible combinations of the nine $|Fm_F\rangle$ states and nine $|R\rangle$ states.

Given these operators, and thus $\hat{M}$, we can write

$$\rho(k_0, x_{\mathrm{f}}) = \hat{M}\rho(k_0)\hat{M}^{\dagger} \qquad (S13)$$

$$= \beta_{k_0} \sum_{m_I m_J} \eta_{m_I m_J} \widehat{M} |k_0 m_I m_J\rangle\langle k_0 m_I m_J| \widehat{M}^\dagger \#$$

$$= \beta_{k_0} \sum_{m_I m_J} \sum_{m_I' m_J'} \sum_{m_I'' m_J''} \eta_{m_I m_J} A^{k_0}_{m_I m_J, m_I' m_J'} A^{k_0 *}_{m_I m_J, m_I'' m_J''} |k_0 m_I' m_J'\rangle\langle k_0 m_I'' m_J''| \qquad \text{(S14)}$$

where $A^{k_0}_{m_I m_J, m_I' m_J'} = \langle k_0 m_I' m_J' | \widehat{M} | k_0 m_I m_J \rangle$. The count rate observed at the detector corresponds to the expectation value of the operator

$$\hat{C} = \sum_{m_I m_J} c_{m_I m_J} |k_0 m_I m_J\rangle\langle k_0 m_I m_J| \qquad \text{(S15)},$$

with $c_{m_I m_J}$ defined in Supplementary Table 2. The purpose of this operator is to take into account the selection of the states by the second hexapole lens. The probabilities of transmission through the lens are $c_{m_I m_J}$. The detector observes all flux passed into it by the hexapole lens, taken into account in $\hat{C}$ by the sum over $m_I m_J$. The count rate is then $C = \mathrm{Tr}\left[\hat{C} \rho(k_0, x_\mathrm{f})\right]$. As B_1 and B_2 are varied, *C* fluctuates, allowing one to obtain curves similar to those shown in Fig. 4 of the manuscript.

By changing B_1 from 0 to 600 gauss and varying B_2 from $-(B_1 - 10$ gauss) to $-(B_1 + 10$ gauss), one can simulate a full-interferometer mode measurement and obtain a 2D plot of the generalized gyromagnetic ratio γ as a function of B_1, as described in the main text. Using 7500 data points in the variation of B_2 produces Supplementary Fig. 5 after Fourier transformation. Overlaying the 36 generalized gyromagnetic ratios expected from $\widehat{H}_\mathrm{R}$ (as defined in the main text) shows that all the observed high intensity features come from the underlying Hamiltonian and match the relation $f_{i,j}(B)$ given in the manuscript. In this calculation, $\sigma^{k_0}_{F' m_F' F m_F} =$

$\delta_{Fm_F,F'm_F'}$ such that the surface is assumed to have no effect other than imparting a change in the propagation direction. The molecules arriving at the surface just prior to this scattering represent an ensemble of linear superpositions of $|Fm_F\rangle$ states (a "mixed state" in density matrix terminology). The details of this mixed state are dependent on the first magnetic field B_1, which varies during a single experiment. The detailed distribution can be determined as:

$$\rho(k_0, x_\mathrm{s}) = \widehat{M'}\rho(k_0)\widehat{M'}^\dagger \qquad (S16),$$

where $\widehat{M'} = \hat{R}_\mathrm{i}\hat{P}_3^{-1}\hat{P}_{\mathrm{B}_1}^{-1}\hat{P}_1^{-1}$ and x_s is the position of the surface.

Clearly many more bands are seen in the model described above than are observed in the experiment (compare Supplementary Fig. 5 and Fig. 4 of the manuscript). The interactions with the sample surface can change the relative amplitudes of the frequency bands, as seen in the measurements presented in Fig. 4b. Further evidence for the sensitivity of the oscillation amplitudes to the surface scattering event can be seen in the calculations shown in Supplementary Fig. 6. The different spectra correspond to different phases of the scattered states. While all the peaks continue to match the transition frequencies (indicated by the red vertical lines), the amplitudes are completely different.

The simulation method presented above allows easy incorporation of various scattering matrices through the operator $\hat{\Sigma}$ and various field angles. It also allows for the study of the state at any point and time in the detector, with little modification to the procedure. This allows for future modeling of time-dependent interactions with the surface and a systematic investigation of molecule-surface interactions. Hence, using this theoretical framework as an interpretation scheme, it should be possible to extend the molecular interference approach presented in the

manuscript to be used as a sensitive molecular beam probe for measuring ultra-fast surface motions, similarly to what was achieved with atomic beams[28].

An alternative time-dependent approach

An alternative, time-dependent approach, which treats the center of mass motion of the molecule classically and the internal (nuclear-spin and rotational) degrees of freedom quantum mechanically, produces similar results to those shown in Supplementary Fig. 6. This simpler method should be appropriate when we want to model static surfaces, i.e. when surface diffusion or surface vibrations on the surface do not change the scattering potential that different Ramsey states encounter. In this approach the nuclear state vector of the molecule at time *t* in the *j*-th (*j*=1,2) arm along its classical path *x*(*t*), is expanded in the complete orthonormal set of eigenvectors, $\left|x_1^{(j)}\right\rangle,...,\left|x_9^{(j)}\right\rangle$, of the Ramsey Hamiltonian under a magnetic field oriented along the *x* (beam) axis, $\mathbf{B}_j = \hat{\mathbf{x}}B_j$:

$$\left|\psi_j(t)\right\rangle = \sum_{n=1}^{9} A_n^{(j)} e^{-i\omega_n(B_j)t} \left|x_n^{(j)}\right\rangle \text{ , } j=1,2 \quad (S17)$$

where $\hbar\omega_n(B_j)$, n=1,...,9 , are the eigenvalues of Ramsey Hamiltonian in the magnetic field B_j . The coefficients $A_n^{(j)}$, n=1,...,9 , determining the state vector in each (*j*=1,2) arm, where the magnetic field $\mathbf{B}_j = \hat{\mathbf{x}}B_j$ is approximately uniform, are obtained by replacing the relatively narrow transition region between two (coarse-grained) neighboring field-zones with a single interface, then imposing continuity boundary conditions on the state vector at the interface. Since the initial magnetic lens polarizes the molecules along the z-axis, the eigenvectors $\left|x_n^{(j)}\right\rangle$

are obtained from the eigenvectors, $\left|z_n^{(j)}\right\rangle$, of the Ramsey Hamiltonian for a magnetic field $\mathbf{B}_j = \hat{\mathbf{z}}B_j$, by the unitary transformation *U*, associated with the rotation of the magnetic field direction to the beam axis in each (*j*=1,2) arm: $\left|x_n^{(j)}\right\rangle = U\left|z_n^{(j)}\right\rangle$. Starting with the initial state vector,

$$\left|\psi\left(t=0\right)\right\rangle = \sum_{m_I m_J} g_{m_I m_J} \left|m_I m_J\right\rangle \quad (S18)$$

, corresponding to the entrance of a molecule to the first arm with amplitudes $g_{m_I m_J}$ for projections (m_I, m_J) of the nuclear spin and rotational angular momentum, respectively, along the z-axis. The coefficients $A_n^{(1)}$, n=1,...,9 , determining the state vector in the first arm ($0 \le t < \tau_1$), are obtained by using the initial (boundary) condition at *t*=0 (*x*(0)=0):

$$\sum_{n=1}^{9} A_n^{(1)} \left|x_n^{1}\right\rangle = \left|\psi_1\left(t=0\right)\right\rangle = \left|\psi\left(t=0\right)\right\rangle \quad (S19).$$

Similarly, the coefficients $A_n^{(2)}$, determining the state vector in the second arm ($\tau_2 \le t < \tau_3$, $\tau_1 < \tau_2$) are obtained by imposing the boundary condition at the entrance time τ_2:

$$\sum_{n=1}^{9} A_n^{(2)} e^{-i\omega_n(B_2)\tau_2} \left|x_n^{(2)}\right\rangle = \left|\psi_2\left(\tau_2\right)\right\rangle = \left|\psi_1\left(\tau_2\right)\right\rangle = \sum_{n=1}^{9} A_n^{(1)} e^{-i\omega_n(B_1)\tau_2} \left|x_n^{(1)}\right\rangle \quad (S20)\ .$$

Now, for the elastic scattering situation discussed above, we may ignore the propagation of the beam over the surface scattering region by assuming $\tau_2=\tau_1=L/v$, $\tau_3=2L/v$, where *L* is the length of a single arm and *v* is the classical molecular velocity, and write the state vector at the exit point, before entering the detecting region, as:

$$\left|\psi_2\left(2L/v\right)\right\rangle = \sum_{n=1}^{9} A_n^{(2)} e^{-i\omega_n(B_2)(2l/v)} \left|x_n^{(2)}\right\rangle \quad (S21).$$

Finally, as the count rate at the detector, located at the end of the beam line, depends on the probability $c_{m_I m_J}$ for a molecule with projections (m_I, m_J) to pass through the second magnetic lens, and on the probability for the molecule to have a velocity *v*, the detected signal (DS) is given by:

$$DS = \sqrt{\pi \Delta v} \int dv e^{-(v-v_0)^2/(\Delta v)^2} \sum_{m_I m_J} c_{m_I m_J} \left|\left\langle m_I m_J \middle| \psi_2\left(2L/v\right)\right\rangle\right|^2 \quad (S22),$$

where v_0 is the center of the Gaussian velocity distribution (=1436 m s^{-1}) and Δv is the distribution width (=4% of v_0). This formula, with the values of the parameters mentioned above, and the distribution functions given in Supplementary Table 2, has been used in the calculations presented in Figs. 2C, 2D and in Supplementary Fig. 7.

Supplementary Note 4. Converting the second arm into a flux detector using a depolarizing B_2 field.

Ideally, one could remove the analyzer hexapole magnet (second magnetic lens) from the setup to convert it into a flux detector, and insert it back into the beam line when full interference measurements are required. In practice, it would be extremely time consuming to do this, as realigning the analyzer magnet with the molecular beam is a complex and lengthy process. In the measurements presented in this manuscript we used an alternative mode to convert the second arm into a flux detector without removing the magnetic lens. The method is based on complete depolarization of the scattered beam and is described in this section.

In the flux detector measurements presented in Fig. 2 of the manuscript, we scan the first field close to the B_1=0 condition in order to manipulate the quantum state prior to the arrival of the molecules at the sample surface. A simple way to convert the second arm into a flux detector is using a strong uncompensated magnetic field $|B_2|>>|B_1|$. It is possible to qualitatively understand why a large enough uncompensated field results in a flux detecting arm effect using simple arguments. The crucial element which makes the second arm a magnetic quantum state detector is the second hexapole magnet which focuses (and defocuses) the different quantum states differently resulting in a state-dependent transmission probability, c_{m_I,m_J} , into the particle detector as described above. The idea is to use a strong B_2 field in order to randomize the quantum states such that the average projection of the quantum state along the detection quantization axis before entering the second hexapole magnet, becomes independent of its value before the beam entered the second arm. Looking at the expression for the detected signal derived above, we see that the phase of the quantum states can be randomized by a combination of a large enough B_2 field and a large enough velocity spread in the beam. When these two parameters are sufficiently large the detected signal no longer depends on the quantum state of the molecule after scattering, and is simply proportional to the total flux of molecules entering the second arm of the instrument. Thus any dependency of the detected signal on the B_1 value is due to a dependence of the scattering probability on the quantum states of the impinging molecules, allowing us to study the stereodynamic scattering process directly.

In the experiments presented in this manuscript the velocity distribution of the H_2 molecules can be approximated as a Gaussian distribution with a FWHM of 4%. In the measurements presented

in Fig. 2 we used a constant field of B_2=500 gauss to convert the arm into a flux detector. We verified that this value is sufficiently large by checking that the oscillation pattern we measured did not change when we scanned the B_2 value (+- 100 gauss) nor when we rotated the quantization axis of the second magnetic lens by 90°.

Another way of assessing whether B_2=500 gauss is sufficiently large to randomize the phases and convert the second arm into a flux detector, is to calculate the signal using the theoretical derivation described above (see expression for DS). Supplementary Fig. 7a shows a calculation of the detected signal as function of B_1 when B_2=0, where the blue markers are the result for a single velocity in the beam and the black markers are for a beam average using a realistic (4% FWHM) beam spread. In these calculations we did not include any change in the quantum state due to scattering, hence, the oscillations reflect the change in the final quantum state due to effect of the B_1 field. As B_1 increases the mismatch between the absolute strengths of the two fields (B_2=0) becomes larger and the oscillations decay due to the velocity spread. Supplementary Fig. 7b shows a similar simulation when B_2 is set to 500 gauss, here the averaging over the velocity distribution makes the oscillations disappear completely, i.e. the phase is sufficiently randomized and the detection is no longer a function of the quantum state at the entrance to the second magnetic lens.

Finally, we emphasize that when we measure close to the spin-echo condition, $B_1 = -B_2$, (e.g. the full-interferometer mode measurements shown in Fig. 4 of the manuscript) the second arm operates as a magnetic state detector even when the fields are large. The reason for this is that the effect of the large fields and finite velocity distributions is compensated by the reversed evolution of the two fields and the coherence is regained for different velocities in the beam,

similarly to what happens in atom or neutron spin echo experiments[1]. Supplementary Fig. 7c demonstrates this by showing a calculation for the signal when B_2=500 gauss and B_1 is scanning close to the spin echo condition.

Supplementary References:

1. Lee, K. *et al.* Evaluation of a density functional with account of van der Waals forces using experimental data of H 2 physisorption on Cu(111). *Phys. Rev. B* **84,** 193408 (2011).
2. Wijzenbroek, M., Klein, D. M., Smits, B., Somers, M. F. & Kroes, G.-J. Performance of a Non-Local van der Waals Density Functional on the Dissociation of H2 on Metal Surfaces. *J. Phys. Chem. A* **119,** 12146–12158 (2015).
3. Klimeš, J., Bowler, D. R. & Michaelides, A. Chemical accuracy for the van der Waals density functional. *J. Phys. Condens. Matter* **22,** 022201 (2010).
4. Dion, M., Rydberg, H., Schröder, E., Langreth, D. C. & Lundqvist, B. I. Van der Waals Density Functional for General Geometries. *Phys. Rev. Lett.* **92,** 246401 (2004).
5. Lee, K., Murray, É. D., Kong, L., Lundqvist, B. I. & Langreth, D. C. Higher-accuracy van der Waals density functional. *Phys. Rev. B* **82,** 081101 (2010).
6. Kroes, G. Six-dimensional quantum dynamics of dissociative chemisorption of H2 on metal surfaces. *Prog. Surf. Sci.* **60,** 1–85 (1999).
7. Born, M. & Oppenheimer, R. Zur Quantentheorie der Molekeln. *Ann. Phys.* **389,** 457–484 (1927).
8. Kohn, W. & Sham, L. J. Self-Consistent Equations Including Exchange and Correlation Effects. *Phys. Rev.* **140,** A1133–A1138 (1965).
9. Hohenberg, P. & Kohn, W. Inhomogeneous Electron Gas. *Phys. Rev.* **136,** B864–B871 (1964).
10. Diaz, C. *et al.* Chemically Accurate Simulation of a Prototypical Surface Reaction: H2 Dissociation on Cu(111). *Science* **326,** 832–834 (2009).

[1] The spin echo compensation is complete (to first order) in the case of helium atoms and neutrons[29,30] and only partial when working with molecules due to the spin-rotation coupling.[25]

11. Gross, A. Reactions at Surfaces Studied by Ab Initio Dynamics Calculations. *Surf Sci Rep* **32,** 291–340 (1998).

12. Kroes, G.-J. & Somers, M. F. Six-dimensional dynamics of dissociative chemisorption of H2 on metal surfaces. *J. Theor. Comput. Chem.* **04,** 493–581 (2005).

13. Kroes, G.-J. & Díaz, C. Quantum and classical dynamics of reactive scattering of H2 from metal surfaces. *Chem Soc Rev* **45,** 3658–3700 (2016).

14. Busnengo, H. F., Salin, A. & Dong, W. Representation of the 6D potential energy surface for a diatomic molecule near a solid surface. *J. Chem. Phys.* **112,** 7641 (2000).

15. Olsen, R. A. *et al.* Constructing accurate potential energy surfaces for a diatomic molecule interacting with a solid surface:H2+Pt(111) and H2+Cu(100). *J. Chem. Phys.* **116,** 3841 (2002).

16. Kosloff, R. Time-dependent quantum-mechanical methods for molecular dynamics. *J. Phys. Chem.* **92,** 2087–2100 (1988).

17. Pijper, E., Kroes, G. J., Olsen, R. A. & Baerends, E. J. Reactive and diffractive scattering of H2 from Pt(111) studied using a six-dimensional wave packet method. *J. Chem. Phys.* **117,** 5885 (2002).

18. Balint-Kurti, G. G., Dixon, R. N. & Marston, C. C. Time-dependent quantum dynamics of molecular photofragmentation processes. *J. Chem. Soc. Faraday Trans.* **86,** 1741 (1990).

19. Balint-Kurti, G. G., Dixon, R. N. & Marston, C. C. Grid methods for solving the Schrödinger equation and time dependent quantum dynamics of molecular photofragmentation and reactive scattering processes. *Int. Rev. Phys. Chem.* **11,** 317–344 (1992).

20. Light, J. C., Hamilton, I. P. & Lill, J. V. Generalized discrete variable approximation in quantum mechanics. *J. Chem. Phys.* **82,** 1400 (1985).

21. Corey, G. C. & Lemoine, D. Pseudospectral method for solving the time-dependent Schrödinger equation in spherical coordinates. *J. Chem. Phys.* **97,** 4115 (1992).

22. Lemoine, D. The finite basis representation as the primary space in multidimensional pseudospectral schemes. *J. Chem. Phys.* **101,** 10526 (1994).

23. Feit, M. ., Fleck, J. . & Steiger, A. Solution of the Schrödinger equation by a spectral method. *J. Comput. Phys.* **47,** 412–433 (1982).

24. Vibok, A. & Balint-Kurti, G. G. Parametrization of complex absorbing potentials for time-dependent quantum dynamics. *J. Phys. Chem.* **96,** 8712–8719 (1992).

25. Ramsey, N. F. Theory of Molecular Hydrogen and Deuterium in Magnetic Fields. *Phys. Rev.* **85,** 60–65 (1952).
26. Jardine, A. P., Fouquet, P., Ellis, J. & Allison, W. Hexapole magnet system for thermal energy 3He atom manipulation. *Rev. Sci. Instrum.* **72,** 3834 (2001).
27. Wu, H., Sprung, D. W. L., Martorell, J. & Klarsfeld, S. Quantum wire with periodic serial structure. *Phys. Rev. B* **44,** 6351–6360 (1991).
28. Jardine, A. P., Alexandrowicz, G., Hedgeland, H., Allison, W. & Ellis, J. Studying the microscopic nature of diffusion with helium-3 spin-echo. *Phys Chem Chem Phys* **11,** 3355 (2009).
29. Alexandrowicz, G. & Jardine, A. P. Helium spin-echo spectroscopy: studying surface dynamics with ultra-high-energy resolution. *J Phys Cond Matt* **19,** 305001 (2007).
30. Mezei, F. *The principles of neutron spin echo*. **128,** (Springer Berlin / Heidelberg, 1980).
